# *N*^6^-methyldeoxyadenosine directs nucleosome positioning in *Tetrahymena* DNA

**DOI:** 10.1186/s13059-018-1573-3

**Published:** 2018-11-19

**Authors:** Guan-Zheng Luo, Ziyang Hao, Liangzhi Luo, Mingren Shen, Daniela Sparvoli, Yuqing Zheng, Zijie Zhang, Xiaocheng Weng, Kai Chen, Qiang Cui, Aaron P. Turkewitz, Chuan He

**Affiliations:** 1 The State Key Laboratory of Biocontrol, MOE Key Laboratory of Gene Function and Regulation, School of Life Sciences, Sun Yat-sen University, Guangzhou, 510060 China; 2 Department of Chemistry, Department of Biochemistry and Molecular Biology, and Institute for Biophysical Dynamics, The University of Chicago, 929 East 57th Street, Chicago, IL 60637 USA; 3 Howard Hughes Medical Institute, The University of Chicago, 929 East 57th Street, Chicago, IL 60637 USA; 4 Key Laboratory of Green Pesticide and Agricultural Bioengineering, Ministry of Education, Guizhou University, Guiyang, 550025 China; 5 Graduate Program in Biophysics, Department of Chemistry and Theoretical Chemistry Institute, University of Wisconsin, Madison, 1101 Univ. Ave., Madison, WI 53706 USA; 6 Department of Molecular Genetics and Cell Biology, The University of Chicago, 920 East 58th Street, Chicago, IL 60637 USA

**Keywords:** *N*^6^-methyldeoxyadenosine, 6mA, m^6^dA, DNA methylation, Nucleosome, Methyltransferase

## Abstract

**Background:**

*N*^6^-methyldeoxyadenosine (6mA or m^6^dA) was shown more than 40 years ago in simple eukaryotes. Recent studies revealed the presence of 6mA in more prevalent eukaryotes, even in vertebrates. However, functional characterizations have been limited.

**Results:**

We use *Tetrahymena thermophila* as a model organism to examine the effects of 6mA on nucleosome positioning. Independent methods reveal the enrichment of 6mA near and after transcription start sites with a periodic pattern and anti-correlation relationship with the positions of nucleosomes. The distribution pattern can be recapitulated by in vitro nucleosome assembly on native *Tetrahymena* genomic DNA but not on DNA without 6mA. Model DNA containing artificially installed 6mA resists nucleosome assembling compared to unmodified DNA in vitro. Computational simulation indicates that 6mA increases dsDNA rigidity, which disfavors nucleosome wrapping. Knockout of a potential 6mA methyltransferase leads to a transcriptome-wide change of gene expression.

**Conclusions:**

These findings uncover a mechanism by which DNA 6mA assists to shape the nucleosome positioning and potentially affects gene expression.

**Electronic supplementary material:**

The online version of this article (10.1186/s13059-018-1573-3) contains supplementary material, which is available to authorized users.

## Background

DNA modifications play a pivotal role in epigenetic regulation. 5-Methylcytosine (5mC) has been shown to participate in various biological processes as the most characterized DNA epigenetic mark in animals and plants [1]. Another form of DNA modification, *N*^6^-methyldeoxyadenosine (6mA or m^6^dA), was discovered in the genomes of both prokaryotes and eukaryotes more than 40 years ago [2, 3]. In prokaryotes, 6mA is involved in numerous processes such as virus defense, DNA replication, DNA repair, transcription regulation, and transposition of DNA [4]. However, the biological significance of 6mA in eukaryotes remained largely unknown. With newly developed high-throughput sequencing technology and sensitive mass spectrometry [5], 6mA has been recently found to be a potential epigenetic mark in the genomes of both unicellular and multicellular organisms [6–10] and was suggested to exist in vertebrates [11–13].

Distinct distribution patterns of 6mA have been revealed in various organisms, with versatile functional roles proposed [14]. In our previous work, we found a periodic distribution of 6mA in the green alga *Chlamydomonas*, suggesting a potential role of 6mA affecting nucleosome positioning and gene expression [6]. The periodic distribution pattern has also been discovered in another eukaryotic organism [15]. Specifically, the genomic locations of the nucleosome and 6mA are anti-correlated, with 6mA marking linker regions between nucleosomes. Although the presence of 6mA has been reported in diverse eukaryotes, genomic distributions and functional implications vary in different species [14, 16]. So far, the periodic pattern around transcription start site (TSS) and the association with nucleosome positioning are exclusively reported in two unicellular organisms: *Chlamydomonas* and *Tetrahymena* [2, 15, 17], raising the question of how pervasive this interesting feature is in eukaryotes. In this study, we used *Tetrahymena* as our model system and explored the periodic distribution pattern of 6mA in vivo and in vitro, suggesting a more conserved functionality of 6mA on nucleosome positioning in eukaryotes.

Organized nucleosome positioning is crucial for gene expression [18]. Constitution of a nucleosome array is directed by both the underlying DNA and chromatin remodelers [19, 20]. Nucleosome formation intrinsically disfavors certain DNA sequences in vitro, especially poly(dA-dT) sequences, suggesting the positioning of nucleosomes could be significantly affected by DNA sequences [21, 22]. The nucleosome locations can even be predicted based on genomic DNA sequences [22, 23]. In vitro nucleosome assembly successfully rebuilt the nucleosome-free region (NFR) around TSS in multiple systems, including yeast and human; however, the phased positioning of nucleosome with periodic pattern can barely be recapitulated [24]. Based on the anti-correlated 6mA nucleosome pattern in both green alga and *Tetrahymena*, we hypothesize that the DNA 6mA methylation could also direct nucleosome positioning. Indeed, in vitro nucleosome assembly assay faithfully rebuilds the nucleosome arrays on native *Tetrahymena* genomic DNA but not on unmethylated DNA. We further used model DNA bearing 6mA modification to perform in vitro nucleosome assembly and measured the 6mA abundance of nucleosome-protected regions versus unprotected regions. We found the nucleosome-protected regions contain much less 6mA than unprotected regions, reinforcing our hypothesis that nucleosome wrapping avoids 6mA-containing DNA in a species-independent manner. Computational simulation indicates DNA flexibility can be modulated by a single 6mA site, which can further affect nucleosome positioning. By homology searching, we identified a MTA70 family protein, TAMT-1 (*Tetrahymena* deoxyadenosine methyltransferase-1), which methylates adenine in the ApT sequence context as demonstrated through an in vitro protein reactivity assay. Knockout of TAMT-1 reduced the total 6mA level and notably altered the transcriptome pattern. Together, we propose that 6mA can direct nucleosome positioning as a DNA marker, which subsequently affects gene expression in an epigenetic way.

## Results

### Determining the 6mA methylome in *Tetrahymena*

**Fig. 1:**
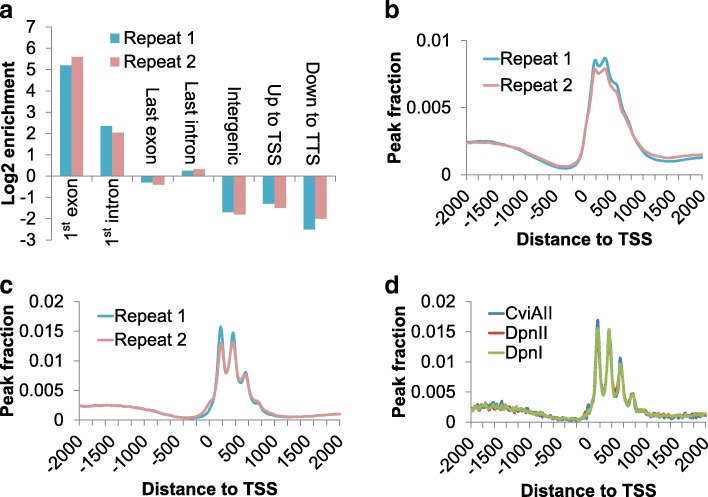
Identification of 6mA in the genome of *Tetrahymena*. **a** Genomic components of genome-wide 6mA distribution. The enrichment score was calculated as the proportion of 6mA peaks dividing the proportion of the attributed genomic component occupying the entire genome. **b** 6mA profile revealed by 6mA-IP-seq. 6mA peaks were identified by comparing reads from IP to input and aligned to the flanking 2 kbp region of TSS. Two biological replicates were performed. **c** 6mA profile revealed by 6mA-CLIP-exo. Two biological replicates were performed. Peaks were aligned to TSS and accumulative distribution was depicted similarly to **b**. **d** 6mA sites revealed by 6mA-RE-seq at single-base resolution. Parallel experiments were performed on the same sample by using three different methylation-sensitive restriction enzymes (DpnI, DpnII, and CviAII). The accumulative individual 6mA sites were aligned to TSS region and depicted similarly to **b**

To characterize 6mA distribution genome-wide, we performed 6mA-IP-seq on genomic DNA from vegetative *Tetrahymena* cells [6]. After comparing the immunoprecipitated (IP) reads with the sequencing background (input), we found 6mA peaks enriched at the first exons and introns but depleted in intergenic regions (Fig. 1a). By aligning the IP reads to the transcription start site (TSS), we found that 6mA is highly enriched near and after TSS (Fig. 1b), resembling the distribution pattern observed in green alga but not in other eukaryotes (*Caenorhabditis elegans* or *Drosophila* or vertebrates) [5, 14]. To narrow down the 6mA loci, we performed 6mA-CLIP-exo, a more precise method by using exonuclease to constrain the target length [6, 25]. The higher resolution map revealed that 6mA is explicitly located in the ~ 1 kbp downstream region of TSS, with a periodic cycle of ~ 200 bp (Fig. 1c). Motif search indicated that these regions are rich in ApT dinucleotides (Additional file 1: Figure S1a), which agrees with the 5′-N(6mA)TN-3′ motif uncovered previously using nearest neighbor analyses assisted by ^3^H labeling in certain genomic loci [26]. To validate the motif, we performed three separate digestions of genomic DNA by using three methylation-sensitive restriction enzymes (DpnI, DpnII, and CviAII) and conducted 6mA-RE-seq, respectively [6]. The distribution of 6mA sites at single-base resolution, located at distinct sequence contexts in the three experiments, agreed with each other, clearly showing a periodic phasing pattern downstream of TSS (Fig. 1d). The flanking region besides restriction sites did not show any consensus sequence, confirming that the ApT dinucleotides are the core sequence for 6mA methylation in *Tetrahymena* (Additional file 1: Figure S1b).

### 6mA and nucleosomes are anti-correlated in vivo

**Fig. 2:**
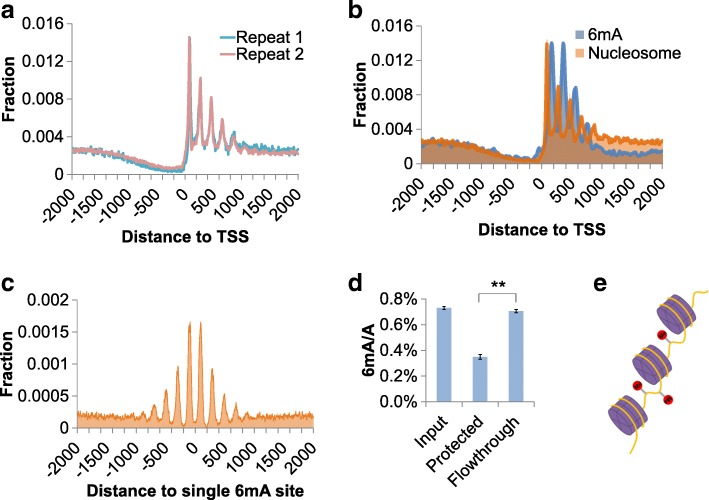
*Tetrahymena* 6mA sites and nucleosome positions are anti-correlated. **a** Nucleosome positioning around TSS. The centers of nucleosomes are predicted and aligned to the 2 kbp region of each TSS. The counts of nucleosomes in each position are normalized to the total counts in the entire region. Two biological replicates were compared. **b** Anti-correlation pattern of 6mA and nucleosome at the downstream regions of TSS. **c** Nucleosome positioning around individual 6mA sites. The nucleosome occupancies around each 6mA site were accumulated and plotted. **d** Total 6mA levels of nucleosome-protected genomic DNA versus input DNA and unprotected regions. Error bars indicate mean ± s.d. of three technical replicates, each measured in duplicates. **p < 0.01 by Student’s *t* test. **e** Illustration of the anti-correlation distribution model. Purple cylinders wrapped with yellow curves represent nucleosomes and DNA. Red circles represent 6mA modifications on DNA

The periodic distribution of 6mA prompted us to check the positions of nucleosomes, which are known to exhibit similar periodic patterns in various eukaryotes [27]. We performed MNase-seq to depict the genome-wide position of the nucleosome in *Tetrahymena* [28]. The MNase-digested chromatin DNA showed explicit ~ 150 bp band, which agreed with the length of DNA wrapping on nucleosome in other known eukaryotic organisms (Additional file 1: Figure S2). By aligning the predicted nucleosome centers to TSS, we found a phasing pattern of nucleosome distribution with ~ 200 bp periodicity. However, unlike other organisms such as yeast or human, the nucleosome-free region is much wider in the upstream of TSS, whereas nucleosome arrays are mainly located downstream of TSS (Fig. 2a). Interestingly, the phasing is exactly opposite of the phasing of 6mA in the downstream regions of TSS (Fig. 2b), indicating an anti-correlated relationship. To investigate the extraordinary pattern in higher resolution, we depicted the accumulated distribution of nucleosome-protected DNA in the vicinity of each individual 6mA sites identified by 6mA-RE-seq and found that 6m sites were significantly disfavored by nucleosome (Fig. 2c). To further validate the lack of nucleosome from 6mA-containing genomic DNA, we performed UHPLC-QQQ-MS/MS to evaluate the 6mA levels of nucleosome-protected DNA fragments versus linker DNA regions. We found that the nucleosome-protected DNA was depleted of 6mA compared to linker regions or input total DNA (Fig. 2d). Together, these results reveal that locations of 6mA and nucleosome are anti-correlated in the genome of *Tetrahymena* (Fig. 2e).

### 6mA directs nucleosome positioning in vitro

**Fig. 3:**
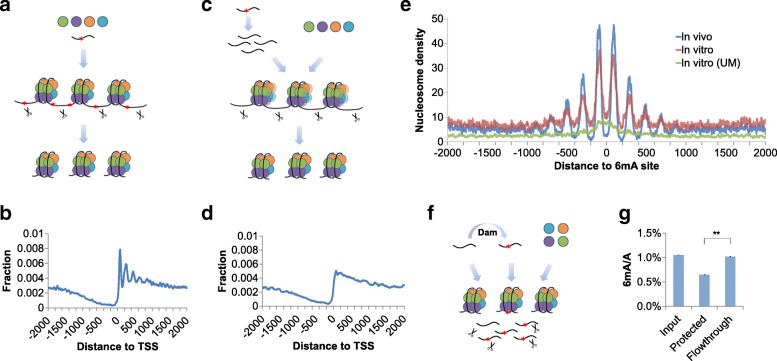
6mA directs nucleosome positioning in vitro. **a** Scheme of in vitro nucleosome assembly on native genomic DNA. Histone H2A, H2B, H3, and H4 are represented as different color balls. DNA is represented as dark curves. The red stars on DNA represent 6mA modification. Scissors represent endonuclease MNase which preferentially digests linker DNA. **b** The sequencing profile of nucleosomes assembled on native genomic DNA. Nucleosome centers were predicted and aligned to the flanking 2 kbp region of each TSS, and the accumulative occupancy was calculated and plotted around TSS. **c** Scheme of in vitro nucleosome assembly on unmethylated genomic DNA. Unmethylated DNA was acquired from whole genome amplification (WGA) from 5 ng genomic DNA. **d** The sequencing profile of nucleosomes assembled on unmethylated genomic DNA from WGA. Nucleosome centers were predicted and aligned to the flanking 2 kbp region of each TSS, and the accumulative occupancy was calculated and plotted around TSS. **e** Comparison of nucleosome distributions around single 6mA sites from three independent conditions: in vivo, native nucleosome profile; in vitro, nucleosome assembly on genomic DNA; and in vitro (UM), nucleosome assembly on unmethylated DNA. **f** Scheme of in vitro nucleosome assembly on model DNA. Unmethylated DNA was mixed with Dam-treated DNA at 1:1 ratio. **g** 6mA level of nucleosome-protected model DNA versus input DNA and unprotected regions. After in vitro nucleosome assembly followed by MNase digestion, UHPLC-QQQ-MS/MS was used to measure the 6mA abundance of input DNA, DNA regions resisting MNase digestion (nucleosome protected), and digested DNA (flowthrough). Error bars indicate mean ± s.d. of three technical replicates, each measured in duplicates. **p < 0.01 by Student’s *t* test

A simple mechanism to explain the anti-correlated 6mA-nucleosome pattern is that the corresponding methylation machinery favors the nucleosome-free region. However, the large NFRs upstream of TSS and downstream of transcription terminal site (TTS) are also depleted of 6mA (Fig. 2b, Additional file 1: Figure S3a). Conversely, 6mA may direct the nucleosome positioning by affecting DNA properties. To verify this assumption, we extracted the native *Tetrahymena* genomic DNA and performed in vitro nucleosome assembly. Purified recombinant histone H2A, H2B, H3, and H4 were mixed with genomic DNA at high NaCl concentration. By gradually decreasing the salt concentration, the histone octamers were generated and genomic DNA started to wrap around the recombinant histone octamers to form nucleosomes. MNase endonuclease was used to digest the linker sequences, leaving the nucleosome-protected portion for high-throughput sequencing (Fig. 3a). Interestingly, previous studies in other organisms such as yeast and *Drosophila* could only reconstitute the NFR surrounding TSS by performing in vitro nucleosome assembly but not the uniform nucleosome positioning. It is worth noting that those organisms previously employed are believed to lack 6mA or have a minor amount below the detection threshold. Adding chromatin remodelers helps to reconstruct the phased nucleosome arrays [20]. Our in vitro nucleosome assembly assay almost perfectly imitated the native nucleosome array downstream of TSS in *Tetrahymena*, without the use of any auxiliary factor (Fig. 3b). These results suggested that 6mA could assist nucleosome positioning in the genome of *Tetrahymena*.

To rule out the potential DNA sequence contributions to nucleosome positioning, we computationally predicted the nucleosome occupancy based on the genomic DNA sequence. As expected, the prediction recapitulated the NFR upstream of TSS and enrichment of nucleosome downstream of TSS but did not show any phased pattern (Additional file 1: Figure S3b). Next, we used Phi29 DNA polymerase to do unbiased whole-genome amplification (WGA) with a small amount of native genomic DNA (~ 5 ng) (Additional file 1: Figure S3c), which was followed by nucleosome assembly using the amplified DNA without methylation due to the dramatic dilution of the native genomic DNA (Fig. 3c, Additional file 1: Figure S3d). Although we could obtain clear ~ 150 bp bands after MNase digestion (Additional file 1: Figure S3e), many fewer nucleosome peaks could be identified by sequencing, suggesting that the nucleosome positioning is randomized on the unmethylated DNA substrate. By aligning the predicted nucleosome locations to gene promoters, we only found NFRs upstream of TSS, without detecting uniform arrays downstream of TSS (Fig. 3d). To compare the nucleosome positions relative to single 6mA sites, we depicted the accumulated nucleosome distribution around specific loci which had been identified to be methylated inside cells. Similar to the in vivo distribution, nucleosomes assembled on extracted genomic DNA exhibit the same periodic pattern, with a significant reduction at 6mA sites. Furthermore, nucleosomes assembled on unmethylated DNA distribute equally along the 6mA flanking regions, even with a slight enrichment at the adjacent area (Fig. 3e), presumably reflecting the sequence composition which in turn partially affects nucleosome positioning [22, 23].

To generalize our observation from *Tetrahymena* genomic DNA, we performed nucleosome assembly on DNA encoding a nucleosome positioning element. This 208 bp synthesized sequence is derived from the *Lytechinus variegatus* (sea urchin) 5S rDNA, which contains one possible binding site for a nucleosome octamer. A single GATC motif is present in the sequence (see the “Methods” section). After the treatment of Dam methylase, we obtained methylation at the GATC site as validated by UHPLC-QQQ-MS/MS (Additional file 1: Figure S3f). Then, we mixed unmethylated and methylated DNA at 1:1 ratio (Fig. 3f). After nucleosome assembly and MNase digestion, we used UHPLC-QQQ-MS/MS to measure the 6mA level of nucleosome-protected DNA versus the flow-through. The protected portion shows much lower 6mA level than the input or the flow-through portion (Fig. 3g), indicating a single 6mA site is sufficient to alter the preference of DNA wrapping on the nucleosome.

### 6mA modulates DNA flexibility

**Fig. 4:**
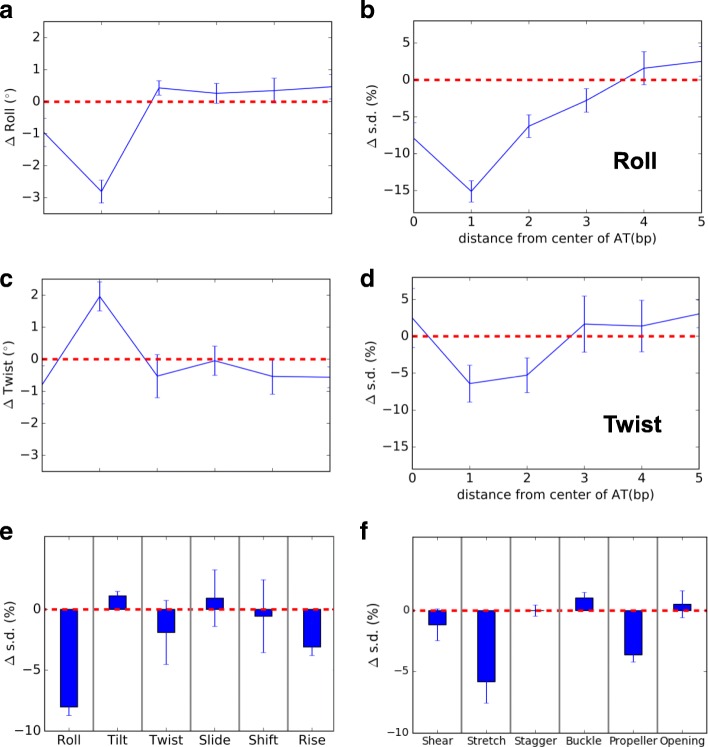
Molecular dynamics simulations of unmodified and 6mA modified DNA. The change after 6mA modification in the mean value of roll (**a**) and standard deviation (s.d.) (**b**) as a function of distance from the center of the modification site. The values are averaged over upstream and downstream directions. **c**, **d** The same as in **a** and **b** but for twist. The average changes after 6mA modification in the s.d. of the inter-base pair (**e**) and intra-base pair (**f**) structural parameters. The values are averaged over 3 bp centered at the modification site. The error bars in all panels are ± 1 standard error of the mean

A change in structural properties of DNA can influence the positioning of nucleosomes. Molecular dynamics simulation was used to investigate the influence of 6mA on DNA structural flexibility. The unmodified and 6mA modified dsDNA in the simulations were compared in terms of inter-base pair (roll, tilt, twist, slide, shift, and rise) and intra-base pair (shear, stretch, stagger, buckle, propeller, and opening) structural parameters; roll and twist were found to be closely related to DNA bending [29]. 6mA modification was found to change the average roll and twist by up to 3° in adjacent positions (Fig. 4a–d), which indicated a change of DNA curvature. 6mA modification decreased the fluctuation of roll and twist by up to 15% and 7%, respectively (Fig. 4b, d). The influence on structural parameters propagated to about 3 bp on each side of the modification site (Additional file 1: Figures S4 and S5). The fluctuation of other parameters of adjacent positions on average was generally reduced except for tilt, slide, buckle, and opening (Fig. 4e, f). The simulations indicated that 6mA modification changes the curvature and rigidifies dsDNA structure, which disfavors nucleosome wrapping. Compared to the effect of 5mC [30] on dsDNA stiffness, the 6mA modification exerts a slightly larger impact.

### TAMT-1 is a 6mA methyltransferase in *Tetrahymena*

We next investigated potential methyltransferase(s) which could be responsible for 6mA methylation in *Tetrahymena*. Previous studies have predicted the existence of multiple DNA 6mA methyltransferases in several eukaryotic organisms, most of which can be attributed to three major families of Dam-like, DNMT, and MTA70 [16]. Among them, the MTA70 family is evolved from MunI-like bacterial DNA 6mA methyltransferase and is widely conserved as RNA m^6^A methyltransferases, including IME4 in yeast, METTL3 in human, and the reported DNA 6mA methyltransferase DAMT-1 in *C. elegans*. The *Tetrahymena* genome encodes two genes belonging to this family, *TTHERM_00136470* and *THERM_00388490*. Both of these genes consist of a MTA70 domain and a ZZ-type zinc finger domain for potential DNA binding. Sequence alignment and phylogenetic distribution analysis of the MTA70 family suggested that these two proteins might be primary candidates for DNA 6mA methylation in *Tetrahymena* (Additional file 1: Figure S6a, b).

**Fig. 5:**
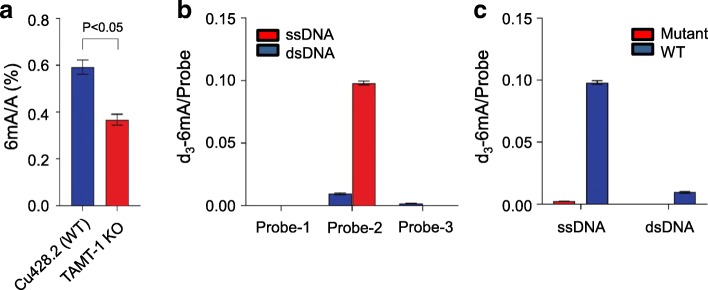
TAMT-1 is a methyltransferase for *N*^6^-deoxyadenosine methylation in *Tetrahymena*. **a** UHPLC-MS/MS quantification of 6mA level in wide-type and TAMT-1 knockout cells. Error bars indicate mean ± s.d. of three biological replicates, each measured in duplicates. **b** In vitro methyltransferase activity of TAMT-1 was tested using different DNA probes (numbered 1–3) with the consensus sequence of CATG, GATC, and random AT. The methylation yields were calculated by the molar ratio of d_3_-m6A to digested probes. Error bars indicate mean ± s.d. of three biological replicates, each measured in duplicate. **c** Mutation of TAMT-1 strongly depleted the methylation activity as detected by UHPLC-MS/MS. Error bars indicate mean ± s.d. of three biological replicates, each measured in duplicate

We created *Tetrahymena* somatic knockout strains of *TTHERM_00136470* and *THERM_00388490* by using homologous DNA recombination, respectively. In these cells, the coding sequence was disrupted with a Neo4 cassette and the knockout efficiency was evaluated by qRT-PCR (Additional file 1: Figure S7a, b). To determine potential functional consequences of these two knockout strains, we extracted genomic DNA from the wild-type strain and knockout strains in the somatic stage and measured the total 6mA level using UHPLC-QQQ-MS/MS. The results show no significant changes in the abundance of 6mA between the *TTHERM_00136470* knockout strain and wild-type control; however, the 6mA level in the *THERM_00388490* knockout strain was significantly reduced (three biological repeats with each having two technical repeats, *p* = 0.0005, *t* test) by 37.8% compared with that of the wild-type control (Fig. 5a), indicating that this gene is at least partially responsible for DNA 6mA methylation in *Tetrahymena*. We thus renamed the gene *TAMT-1* as *Tetrahymena* deoxyadenosine methyltransferase-1.

We then sought to determine whether TAMT-1 could catalyze 6mA methylation in vitro. The low expression level of recombinant TAMT-1 and degradation of purified protein hindered us to get enough amounts of proteins to perform the entire enzymatic kinetic assay. Instead, we cloned and purified the catalytic domain of TAMT-1 consist of the MTA70 and the ZZ-type zinc finger domain (Additional file 1: Figure S7c). DNA probes with consensus sequences of CATG (Probe-1), GATC (Probe-2), and random AT (Probe-3) in double- and single-stranded forms were incubated with purified TMAT-1 separately. S-(5′-adenosyl)-l-methionine-d3 (d_3_-SAM) was used as the cofactor for accurate UPLC-QQQ-MS/MS quantification. We calculated the methylation yields by the molar ratio of newly formed d_3_-6mA to each digested DNA probe (Additional file 1: Figure S7d). The result showed that the catalytic domain of TAMT-1 exhibited no activity with Probe-1 and Probe-3, whereas it showed considerable methyltransferase activity towards Probe-2 with a GATC motif in double- and single-stranded DNA. We further measured the time course of the reaction between the catalytic domain of TAMT-1 and Probe-2 to further confirm the enzyme activity (Additional file 1: Figure S7e). In particular, the enzyme showed a strong preference for the single-stranded DNA probe (tenfold) compared to the corresponding duplex DNA (Fig. 5b). To exclude the possibility that the methylation activity was caused by the contamination of *Escherichia coli* methyltransferase(s) during purification, and further confirm that the methylation activity was directly mediated by TAMT-1, we mutated the methylation signature motif DPPW and highly conserved amino acid residues E111 and K183 which play important roles in catalysis [31]. The result showed that mutations of DPPW to APPA, together with E111A and K183A, significantly decreased the in vitro methylation activity, suggesting that the methylation activity was mediated by TMAT-1 (Fig. 5c). Taken together, these results confirm that TAMT-1 is a 6mA methyltransferase in *Tetrahymena*.

### Deficiency of 6mA perturbs gene expression

**Fig. 6:**
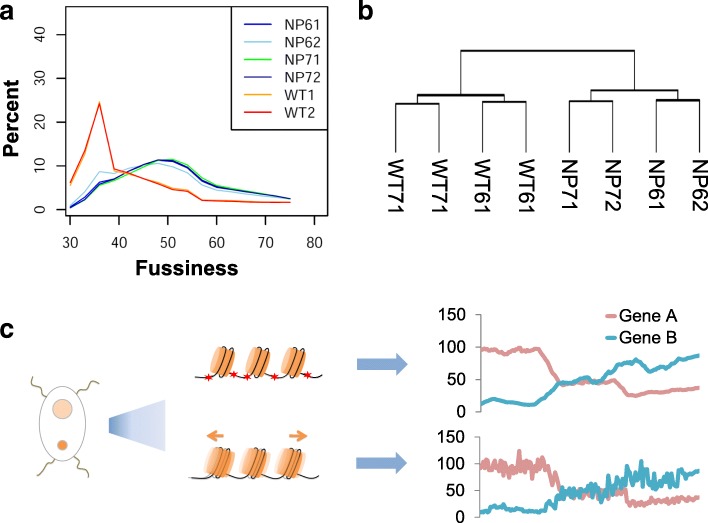
Nucleosome profile and transcriptome change in 6mA defective *Tetrahymena*. **a** Fuzziness of genome-wide nucleosome profile in wild-type (WT1, WT2) and methyltransferase knockout (NP61, NP62, NP71, NP72) cells. Fuzziness is defined as the deviation of nucleosome positions within each unit and is calculated by software DANPOS [32]. Two biological replicates for KO cells were constructed. NP61 and NP62 are two technical replicates for one cell strain, and NP71 and NP72 are two technical replicates for another cell strain. The smaller score represents better well-phased nucleosomes. **b** Clustering analysis of transcriptome for WT (WT61/WT62 and WT71/WT72 are two wild-type strains; each strain contains two technical replicates) and KO (NP61/NP62 and NP71/NP72 are two KO strains; each strain contains two technical replicates) cells. WT and KO cells are distinctly separated with a large difference. **c** Scheme for the model that 6mA stabilizes gene expression. In WT, 6mA is positioned to constrain nucleosome positioning which regulates gene expression. In KO, the level of 6mA significantly reduced. Nucleosomes lacking of 6mA constraints tend to be fuzzier which leads to larger transcriptional fluctuation

To study the potential effects of 6mA deficiency, we performed 6mA-IP-seq to access methylation changes between KO and wild-type cells. Interestingly, the 6mA peak counts did not change much. We reasoned that the methylation intensity was evenly decreased at most sites, but the genome-wide methylation pattern was maintained. To further validate the effect of TMAT-1 KO, we randomly selected ten genomic loci harboring 6mA sites and three loci without 6mA as the negative control. 6mA-IP-qPCR showed the 6mA level of eight of the ten 6mA sites in KO strain decreased significantly compared to that in WT strain (two biological repeats with each having three technical repeats, *p* < 0.05, *t* test), indicating the reduced 6mA modification on those tested loci by disruption of TMAT-1 (Additional file 1: Figure S8a, Additional file 2: Table S1). Then, we used MNase-seq to profile the nucleosome pattern of the KO cells. We expected that decreased intensity of 6mA at most sites could affect nucleosome positions observed in the wild-type strain. To quantify how well the nucleosomes localized in an array, we used a fuzziness score to represents the average level of genome-wide nucleosome positioning [32]. Indeed, we found that the genome-wide nucleosome profile was significantly altered in the KO cells compared to WT cells, with a dramatically elevated overall fuzziness score (Fig. 6a), and the sharpness of the nucleosome positioning array around TSS is attenuated in KO cells (Additional file 1: Figure S8b). Nucleosome positioning is known to interact with transcription machinery and affect gene expression in complex ways; well-phased nucleosome arrays safeguard the robustness of transcription [27, 33, 34]. To verify our hypothesis, we performed RNA-seq to measure the levels of transcripts in KO cells versus WT cells. We identified significant alteration of global transcriptome in KO cells (Fig. 6b), with hundreds of genes being significantly up- or downregulated (Additional file 1: Figure S8c). These observations led us to hypothesize that 6mA directs nucleosome positioning, which in turn stabilizes gene expression; reducing 6mA disrupts normal nucleosome patterns and induces transcriptome-wide changes (Fig. 6c).

## Discussion

As a ciliated protozoan, *Tetrahymena* diverged from vertebrates or green alga more than two billion years ago [35–37]. The analogous distribution patterns of 6mA in green alga and *Tetrahymena* as well as the most recent discoveries in fungi suggest conserved biogenesis pathways and functions of this DNA modification inherited from an ancient common ancestor [10]. The methylation machinery could also be acquired independently by convergent evolution.

We performed in vitro nucleosome array assembly using recombinant histones and model DNA mixed with Dam-methylated DNA from an unrelated organism. Mass spectrometry showed that nucleosomes intrinsically disfavor methylated DNA. Though in vitro experiments showed that 6mA directs nucleosome assembly without any other auxiliary proteins, 6mA may recruit partner proteins in vivo which could reinforce the chromatin architecture. We further showed that the mutual repulsion effect between nucleosome and 6mA is independent of species or DNA sequence. Because 6mA appears to be a prevalent DNA modification in many species including mammals, the effect of 6mA on directing nucleosome positioning in *Tetrahymena* may also applies to other organisms as this appears to be an intrinsic biophysical property associated with 6mA in DNA.

How could 6mA repel nucleosome wrapping? It has been shown that the presence of methylation could rigidify the double-stranded DNA structure [30]. The methylation can reinforce the base stacking along the duplex. Another source of energy comes from reduced solvation penalty when 6mA is packed along the lineal duplex. A potential bending of the 6mA-containing duplex DNA such as wrapping around the nucleosome would expose the hydrophobic methyl group to the solvent water, inducing negative solvation penalty. The fully methylated 6mA on the opposite strands and clustering of 6mA methylation further amplify these effects, which collectively rigidify duplex DNA.

We identified a potential 6mA methyltransferase, TAMT-1, which demonstrates in vitro activity on ApT dinucleotides. TAMT-1 knockout suppresses 6mA levels in vivo. Only one 6mA methyltransferase has been found in *C. elegans* in the past [7]. Intriguingly, these two candidates belong to the well-conserved MTA70 protein family, which also includes mRNA methyltransferases [38]. The widespread distribution of this protein family may suggest a more extensive presence of 6mA in uncharacterized organisms [5, 16]. By disrupting TAMT-1, we observed a dramatic decline of 6mA levels in living cells. Additional methyltransferases may exist which could explain the remnant 6mA in the KO strain. Nevertheless, the discoveries of 6mA methyltransferases provided us with a useful target to manipulate 6mA and perform more thorough functional studies in the future.

It is worth noting that in certain organisms, Dnmt5 generates dense 5mC clusters which directly disfavor nucleosomes [39]. A similar mechanistic argument with better packing and reduced solvation penalty could explain the effect of 5mC. It is thus very likely that 5mC may also affect nucleosome positioning in more widespread organisms. Interestingly, in our previous study, we discovered that 5mC and 6mA generally tend to avoid each other in the *Chlamydomonas* genome, suggesting complementary roles of different DNA modifications in one organism. *Tetrahymena* provides us with a unique model system to study the interaction between 6mA and nucleosome as it possesses only 6mA but not 5mC, reinforcing our notion that 6mA plays a key role in nucleosome positioning.

Until recently, 5mC and its derivatives were the well-established epigenetic marks in eukaryotic genomes. The recently discovered prevalence and functional implications of 6mA open a new avenue in epigenetic research. In this study, we found that the anti-correlation to nucleosome is a well-conserved feature of 6mA in evolutionary distinct species. Species-independent, 6mA-modified DNA is intrinsically disfavored by a nucleosome. In higher eukaryotes in which 5mC is recognized by reader proteins to suppress gene expression, the presence of 6mA could provide additional tuning of transcription by affecting nucleosome positioning or by resisting bending incurred by various DNA-binding proteins such as transcriptional factors. Overall, our results show the intrinsic properties of the DNA 6mA methylation can significantly impact gene expression and biological outcome in eukaryotes.

## Conclusions

In this study, we reveal the intrinsic repulsion between 6mA and nucleosomes, which contributes to shape nucleosome positioning and affect gene expression. By disrupting the newly identified potential methyltransferase, we observed significant transcriptome changes. We propose that disordered nucleosome positioning caused by 6mA depletion is one of the main reasons underlying the observed phenotypes.

## Methods

### Cell strains and DNA/RNA collection

Cell strain SB210 was obtained from *Tetrahymena* Stock Center (https://tetrahymena.vet.cornell.edu/strains.php) and cultured in proteose peptone (PP) medium (2% proteose peptone, 10 μM FeCl_3_, or 90 μM sequestrene (Fe-EDTA)) at 31 °C. Culture densities were measured using Z1 Coulter Counter (Beckman Coulter). The genomic DNA was extracted by Quick-DNA™ Miniprep Kit (Zymo Research, Cat. No. D3024). Total RNA was extracted by Direct-zol™ RNA MiniPrep (Zymo Research, Cat. No. R2050) and further purified to mRNA by Dynabeads® mRNA Purification Kit (Thermo Fisher Scientific, Cat. No. 61006).

### Generation of TAMT1 knockout strains

Eight hundred sixty-two base pairs of sequence from upstream of *tamt-1* (*TTHERM_00388490*) coding region and a portion of the open reading frame (ORF) (1,139 bp) were amplified and subcloned into the pNEO4 vector flanking the *neo4* cassette with *SacI*/*PstI* and *XhoI*/*KpnI* restriction cutting sites, respectively. The sequences of primers are listed in Additional file 2: Table S2. The construct resulted in the deletion of *tamt-1* genomic region from 0 to 907 bp. The knockout vector was linearized by digestion with *SacI* and *KpnI* then transformed into CU428.1 cells by biolistic transformation following the reported method [40]. To assess the gene disruption, RT-qPCR was performed.

Total RNA was isolated by using RNeasy Plus Mini kit (Qiagen). Five hundred nanograms total RNA were reverse-transcribed into cDNA with PrimeScript™ RT reagent Kit (Takara) and then subjected to qPCR analysis with FastStart SYBR Green Master Mix (Roche) in a Roche LightCycler 96. ATU1 were used as internal control. Relative changes in expression were calculated using the ΔΔCt method. All RT-qPCR primers are listed in Additional file 2: Table S2.

### 6mA-IP-seq

One microgram purified genomic DNA was segmented to ~ 250 bp by sonicator (Bioruptor, Diagenode). Then, DNA segments were end-repaired, 3′-adenylated, and ligated to Illumina adaptors by using NEBNext® DNA Library Prep Kit (NEB, Cat. No. E6040S). 6mA-containing DNA was enriched by antibody immunoprecipitation (SYSY, Cat. No. 202 003) then subjected to NGS (Illumina, HiSeq 2500). 6mA peaks were called by MACS2 by comparing the pull-down reads versus input DNA. Cutoff FDR < 0.01 was set to increase the reliability of 6mA candidates. Detailed protocol can be found in Reference [8].

### 6mA-CLIP-exo

Genomic DNA containing 6mA was first immunoprecipitated following a procedure similar to previously described with 6mA-IP-seq. Target DNA was then covalently cross-linked to the antibody by UV 254 nm irradiation, followed by lambda exonuclease digestion (NEB, Cat. No. M0262S). After adaptor ligation and PCR amplification (18 cycles), DNA library was constructed for NGS (Illumina, HiSeq 2500). Detailed protocol can be found in Reference [8].

### 6mA-RE-seq

Purified genomic DNA was digested by three different restriction enzymes separately (DpnI, DpnII, and CviAII). Digested DNA segments were further sheared to ~ 250 bp by sonicator (Bioruptor, Diagenode). DNA segments were end-repaired, 3′-adenylated, and ligated to sequencing adaptor by using NEBNext® DNA Library Prep Kit (NEB, Cat. No. E6040S). After PCR amplification for 15 cycles, purified DNA library was constructed for NGS (Illumina, HiSeq 2500). Detailed protocol can be found in References [6, 9].

### Nucleosome positioning analysis

The nucleosome positioning is mainly determined by MNase-seq. Cells were lysed, and nuclei were isolated by Nuclei Isolation Kit (Sigma-Aldrich, Cat. No. NUC101). After MNase treatment for 10 min, DNA fragments were purified by DNA Clean & Concentrator Kit (Zymo Research, Cat. No. D4013) and loaded into agarose gel. The ~ 150-bp band was isolated and extracted by Zymoclean™ Gel DNA Recovery Kit (Zymo Research, Cat. No. D4007). DNA library was constructed by NEBNext® DNA Library Prep Kit (NEB, Cat. No. E6040S) according to the standard Illumina DNA library preparation procedures.

The 208-bp model DNA was purchased from NEB (Cat. No. N1202S), which was originated from *Lytechinus variegatus* (sea urchin) 5S rDNA and supposed to contain one nucleosome binding site. The entire sequence has only one GATC site (see below) where the A can be methylated to 6mA by Dam methyltransferase (NEB, Cat. No. M0222S), ACTTCCAGGGATTTATAAGCCGATGACGTCATAACATCCCTGACCCTTTAAATAGCTTAACTTTCATCAAGCAAGAGCCTACGACCATACCATGCTGAATATACCGGTTCTCGTCC*G*(*A*)*TC*ACCGAAGTCAAGCAGCATAGGGCTCGGTTAGTACTTGGATGGGAGACCGCCTGGGAATACCGAATTCCCCGAGGAATTCCAACGAATA

In in vitro nucleosome assembly experiments, purified recombinant human histone H2A and H2B were mixed to generate dimer in advance, as well as histone H3.1/H4 tetramer. Then, 100 pm dimers and 50 pm tetramers were mixed with 50 pm DNA at 2 M NaCl. The salt concentration was sequentially diluted to allow the nucleosome generation on the DNA. Detailed procedures can be found in the EpiMark® Nucleosome Assembly Kit (NEB, Cat. No. E5350S). Assembled DNA/nucleosome component was then digested by MNase, and the protected region was purified for further analysis.

Nucleosome fuzziness is defined as the deviation of nucleosome positions within each unit in a cell population. The loci and fuzziness of nucleosome were calculated by software DANPOS [32].

### Molecular dynamics simulations

The initial atomistic model of an unmodified 33-bp B-DNA fragment was built using w3DNA web interface [41] to the 3DNA software package [42]. The sequence of the fragment was GCTCACCCGCGCCC*AT*GGTGGGAGCCGGAGACG, where the positions of potential N^6^-methylation are in italics. The structure of the modified fragment was obtained by modifying the initial atomistic model using PyMol (http://pymol.org/). Molecular dynamics simulations were carried out using AMBER16 package with graphics processing units [43]. Parmbsc1 force field was used in the simulations [44]. Parameters for N^6^-methyladenosine was adapted from those of RNA N^6^-methyladenosine [45]. The structures were solvated in TIP3P water [46] such that the closest distance between DNA atoms and the truncated octahedral water box edge was greater than 10 Å. The system also contained 150 mM NaCl after neutralizing with Na+ ions. Particle mesh Ewald was used to calculate the electrostatic interactions with a grid spacing of about 1.0 Å. The non-bonded cutoff was 12 Å with the missing long-range van der Waals interactions approximated with a long-range continuum correction [47]. Energy minimization was done by 3000 steps of steepest descent followed by 97,000 steps of conjugate gradient. The DNA was fixed initially and then relaxed to minimize the energy of the entire system. One nanosecond of NPT simulation was used to equilibrate the density of the system. Production simulations were carried out in NVT ensemble at 300 K using Langevin dynamics with a collision frequency of 1 ps^−1^. SHAKE algorithm was used to constrain all bonds involving hydrogen atoms, which allowed an integration step of 2 fs. The trajectories were about 500 ns long for each construct. Conformation snapshots were saved every 20 ps. Structural parameters of DNA were computed using the 3DNA program [42]. The error bars in Fig. 1 and Additional file 1: Figures S1 and S2 are standard errors of the mean calculated using block averaging and conventional error propagation rules.

### Validation of 6mA sites

Input percentage method was used to validate the 6mA site and measure the relative abundance by real-time qPCR; Ct values were used for performing the calculation which consists on evaluating the fold difference between 6mA-IP sample and input. The equation used lies below:

$$ \Delta \mathrm{Ct}\ \left[\mathrm{normalized}\ 6\mathrm{mA}-\mathrm{IP}\right]=\left(\mathrm{Ct}\ \left[6\mathrm{mA}-\mathrm{IP}\right]-\right(\mathrm{Ct}\ \left[\mathrm{Input}\right]-\mathrm{Log}2\ \left(\mathrm{Input}\ \mathrm{Dilution}\ \mathrm{Factor}\right). $$

In this work, the fraction of input saved is 10 μl and the fraction for each 6mA-IP is 70 μl. The IP fraction is seven times to the input fraction. The equation above is as follows: ΔCt [normalized 6mA-IP] = (Ct [6mA-IP] − (Ct [Input] − Log2 (7)). The percentage (Input %) value for each sample is calculated as follows: Input % = 100/2 ΔCt [normalized 6mA-IP]. The “Input %” value represents the enrichment of certain 6mA modification on a specific region. Two replicates are performed and averaged to represent the relative abundance.

## Additional files

Additional file 1: **Figure S1.** Preferred sequence motifs for *Tetrahymena* 6mA sites. **Figure S2.** Agarose gel of MNase digested nuclei. **Figure S3.** Nucleosome assembly favors 6mA-free regions in vitro. **Figure S4.** Changes of mean value after 6mA modification in the intra-base pair (A-F) and inter-base pair (G-L) parameters as a function of distance from the center of the modification site. **Figure S5.** Changes of standard deviation (s.d.) after 6mA modification in the intra-base pair (A-F) and inter-base pair (G-L) parameters as a function of distance from the center of AT. **Figure S6.** Multiple sequence alignment and phylogenetic distribution analysis of MTA70 family. **Figure S7.** Knockout of two methyltransferases in *Tarahymena* and in vitro methylation activity characterization of methytransferaseTAMT-1. **Figure S8.** Effects of *tamt-1* knockout. (DOC 11096 kb)

Additional file 2: **Table S1.** Genomic loci for individual 6mA testing. Table S2. Sequences of primers used for *tamt-1* KO experiment. (DOC 47 kb)
