# Supplementary material for: N6-methyldeoxyadenosine directs nucleosome positioning in Tetrahymena DNA
Source: Genome Biol. 2018 Nov 19;19:200. doi: 10.1186/s13059-018-1573-3 (PMC6245762; doi:10.1186/s13059-018-1573-3)
Supplement: Supplementary file 1 — Figure S1. Preferred sequence motifs for Tetrahymena 6mA sites. Figure S2. Agarose gel of MNase digested nuclei. Figure S3. Nucleosome assembly favors 6mA-free regions in vitro. Figure S4. Changes of mean value after 6mA modification in the intra-base pair (A-F) and inter-base pair (G-L) parameters as a function of distance from the center of the modification site. Figure S5. Changes of standard deviation (s.d.) after 6mA modification in the intra-base pair (A-F) and inter-base pair (G-L) parameters as a function of distance from the center of AT. Figure S6. Multiple sequence alignment and phylogenetic distribution analysis of MTA70 family. Figure S7. Knockout of two methyltransferases in Tarahymena and in vitro methylation activity characterization of methytransferaseTAMT-1. Figure S8. Effects of tamt-1 knockout. (DOC 11096 kb) [file 13059_2018_1573_MOESM1_ESM.doc]

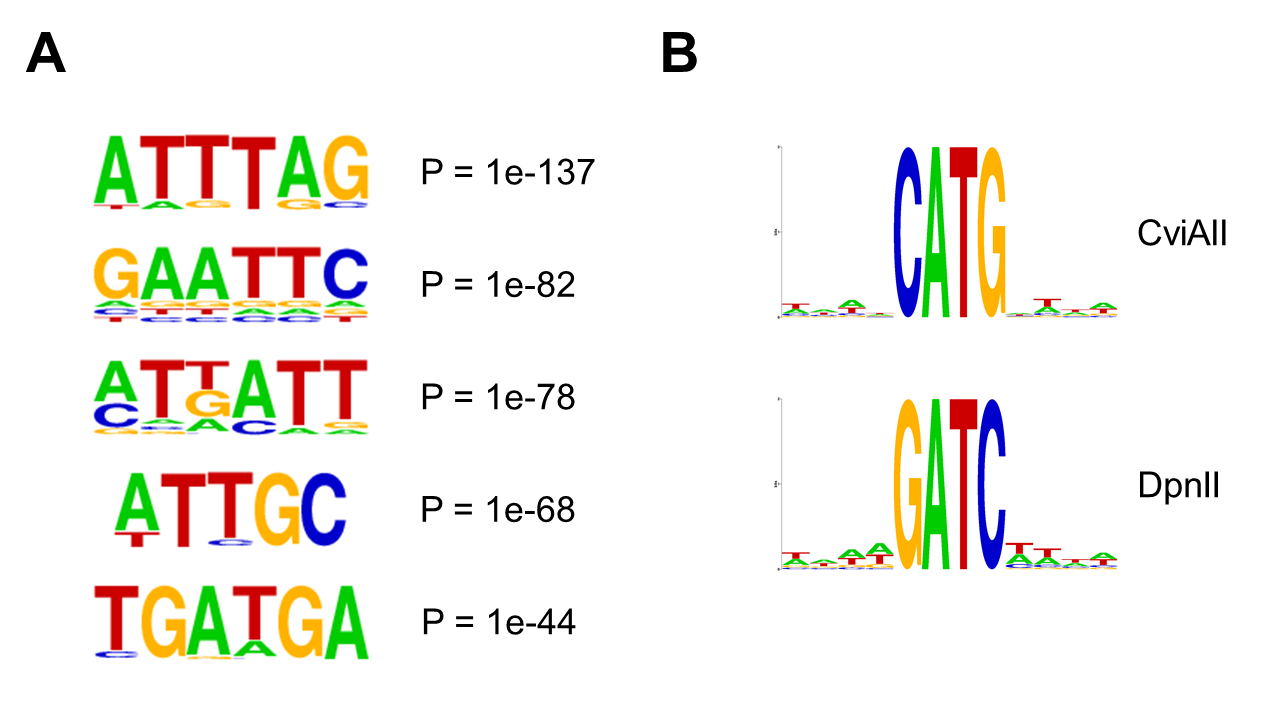


**Figure S1.** Preferred sequence motifs for *Tetrahymena* 6mA sites. (A) Sequence motif discovered in 6mA-IP-seq. Homer program was used to search the consensus sequences in the 6mA peak regions. (B) Sequence logo of the 6mA sites identified by 6mA-RE-seq and the flanking 4 bp regions.


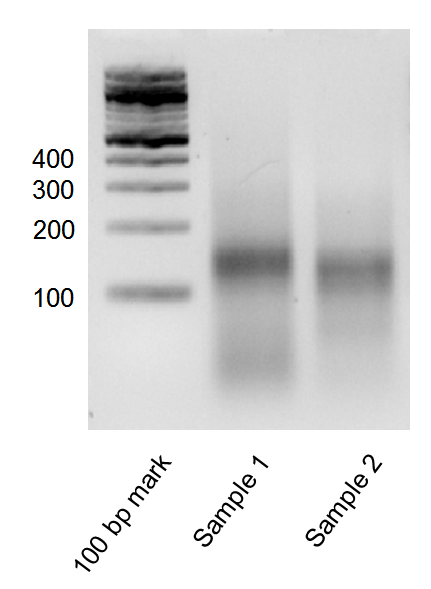


**Figure S2.** Agarose gel of MNase digested nuclei. The bands around 150 bp represent the nucleosome protected regions which resisted MNase digestion.


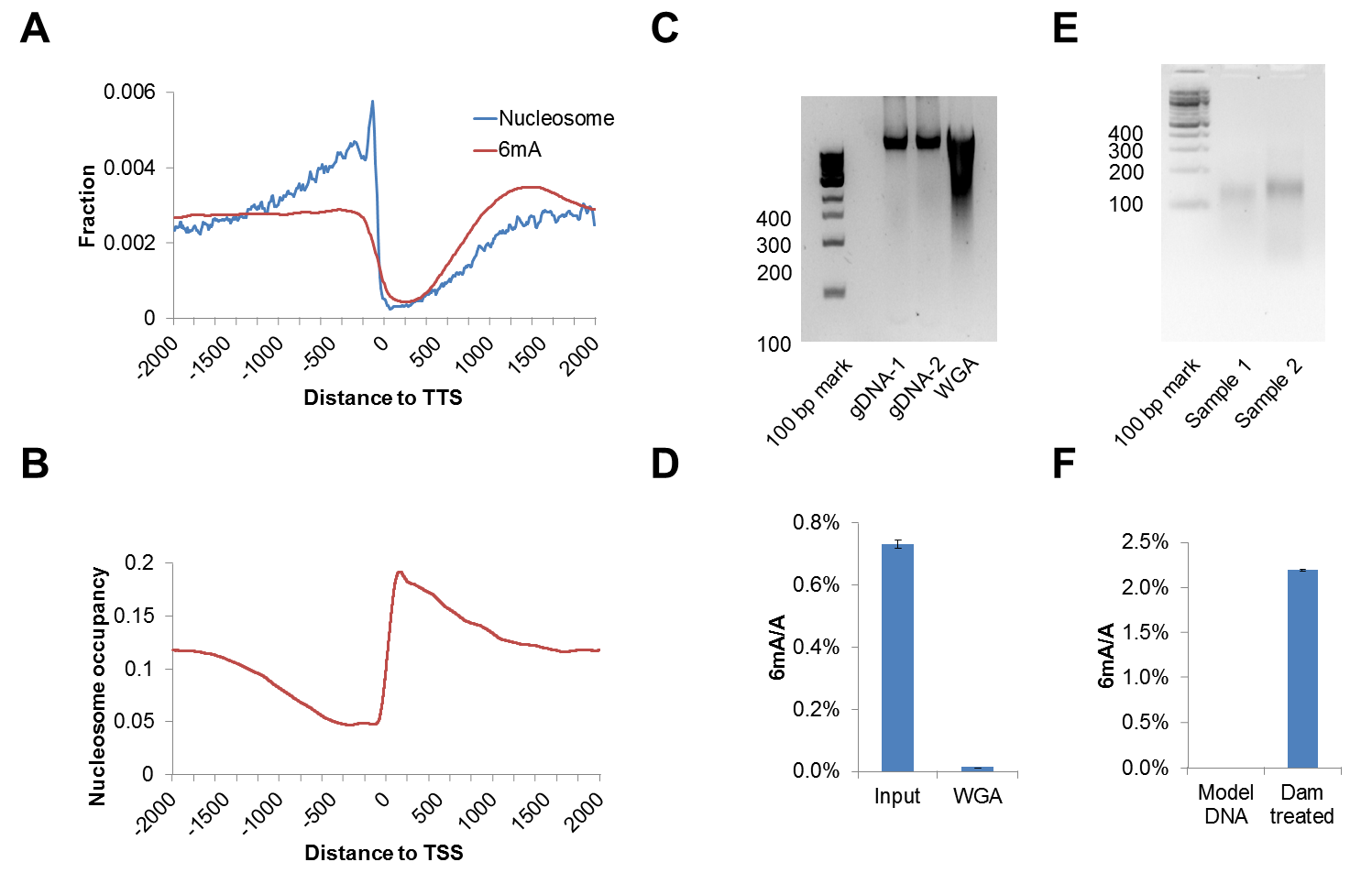


**Figure S3.** Nucleosome assembly favors 6mA-free regions *in vitro*. (A) 6mA abundance decreased following transcription terminal site. (B) Computationally predicted nucleosome occupancy around TSS area. (C) Agarose gel showed the size of genomic DNA and the DNA products from whole genome amplification (WGA). (D) 6mA abundance (6mA/A) quantification by UHPLC-QQQ-MS/MS of native genomic DNA (Input) and whole genome amplified DNA (WGA). After WGA, 6mA was largely diluted comparing to the native genomic DNA. (E) Agarose gel showed ~150 bp bands of MNase digested nucleosome assembly which used WGA DNA as the substrate. (F) 6mA abundance (6mA/A) quantification by UHPLC-QQQ-MS/MS of model DNA and Dam treated DNA which contained one GATC site.


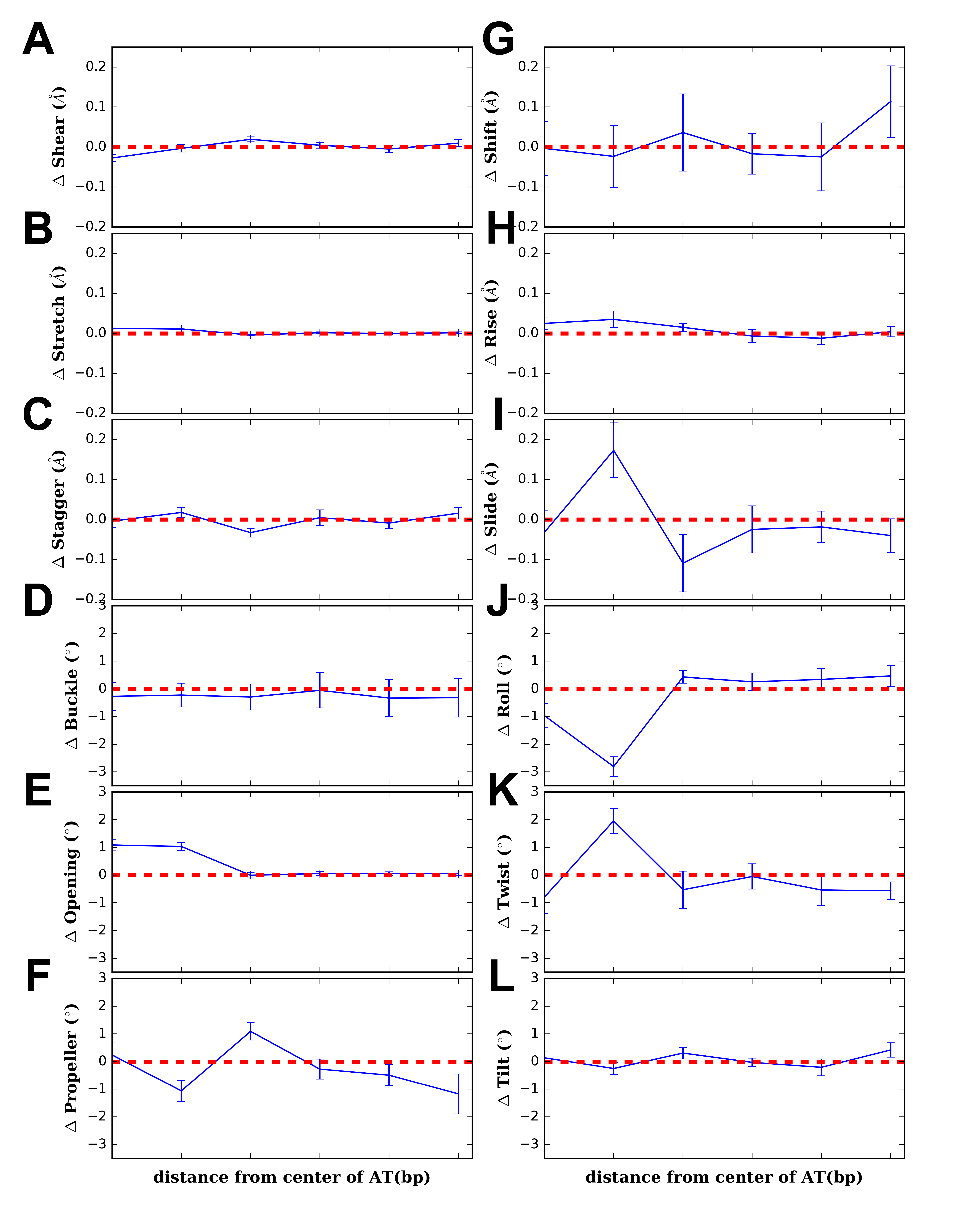


**Figure S4.** Changes of mean value after 6mA modification in the intra-base-pair (A-F) and inter-base-pair (G-L) parameters as a function of distance from the center of the modification site. The values are averaged over upstream and downstream directions. The error bars are +/- one standard error of the mean.


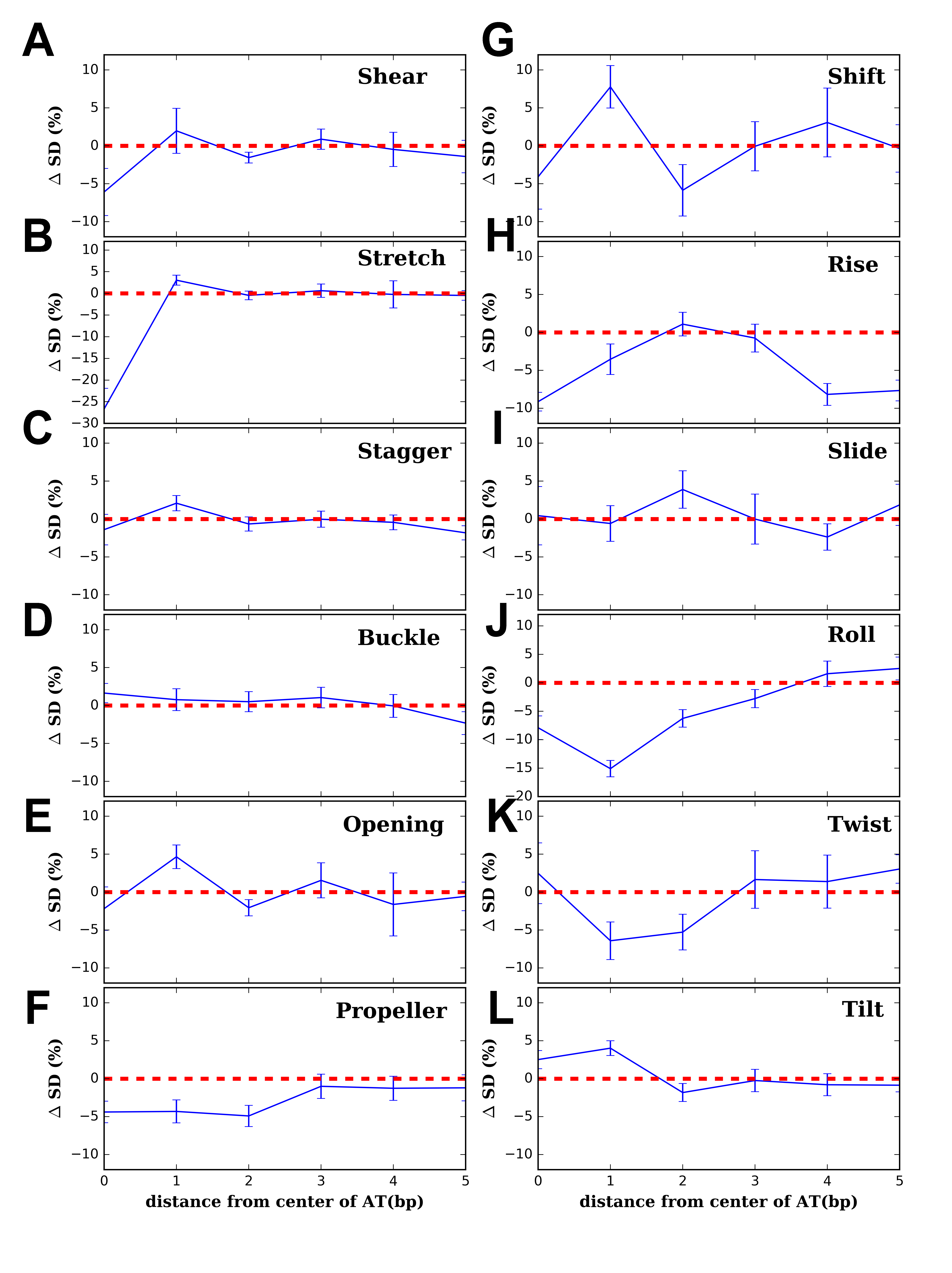


**Figure S5.** Changes of standard deviation (s.d.) after 6mA modification in the intra-base-pair (A-F) and inter-base-pair (G-L) parameters as a function of distance from the center of AT. The values are averaged over upstream and downstream directions. The error bars are +/- one standard error of the mean.


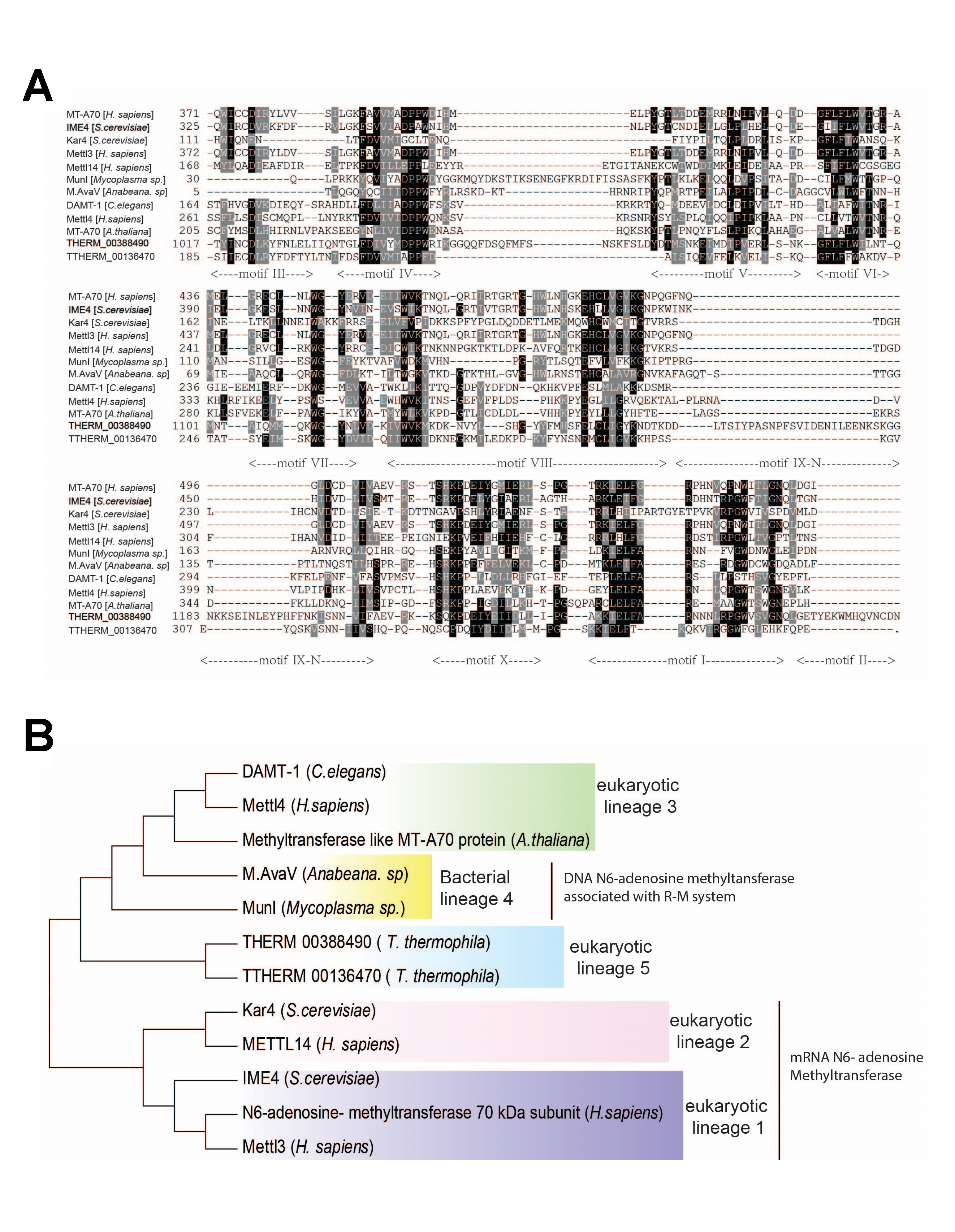


**Figure S6.** Multiple sequence alignment and phylogenetic distribution analysis of MTA70 family. (A) Multiple sequence alignment of two putative *Tetrahymena* candidates was performed with other MTA70 family members using T-coffee and Boxshade programs. Identical and semi-conserved residues are highlighted in black and grey respectively. (B) Phylogeny tree of MTA70 family members in bacterial and eukaryotic lineages.


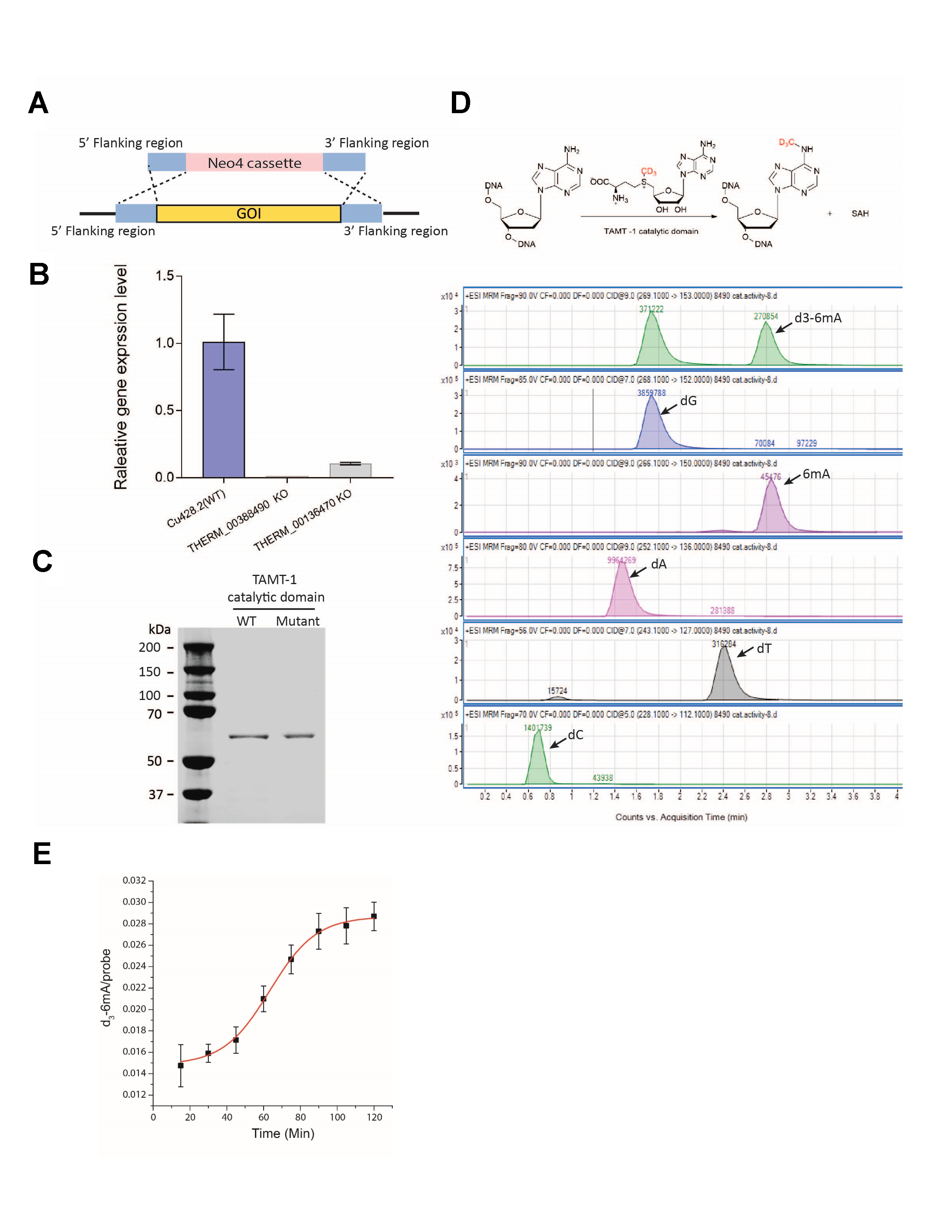


**Figure S7**. Knockout of two methyltransferases in *Tarahymena* and *in vitro* methylation activity characterization of methytransferaseTAMT-1. (A) Strategy to generate knockout cells. The wild-type *TTHERM_00136470* and *THERM_00388490* locus was homologously replaced by Neo4 cassette (pink box), targeted disrupt the gene of interest (yellow box). (B) Knockout efficiency of two *Tetrahymena* candidates was assessed by real-time qRT-PCR. (C) Catalytic domain of TAMT-1 and its mutant were expressed, purified and characterized by SDS-PAGE gel. (D) d3-SAM was used as the cofactor to eliminate the potential contamination of m6A which already bound to the methyltransferase during the purification process. The representative UHPLC-MS/MS spectra illustrates the newly formed d3-m6A as well as the digested nucleotides (dA, dT, dC, dG and 6mA) in probe 2. (E) Time course of methylation catalyzed by TAMT-1 on DNA probe-2. Reactions (50 μl) containing 50 nM TAMT-1, 200 nM DNA probe and 4 μM d3-SAM were incubated at 30 °C. The methylation yields were calculated on the basis of the molar ratio of d3-6mA to probe, measured by UHPLC-MS/MS. Error bars indicate mean ± s.d., n = 4 (two biological replicates × two technical replicates).


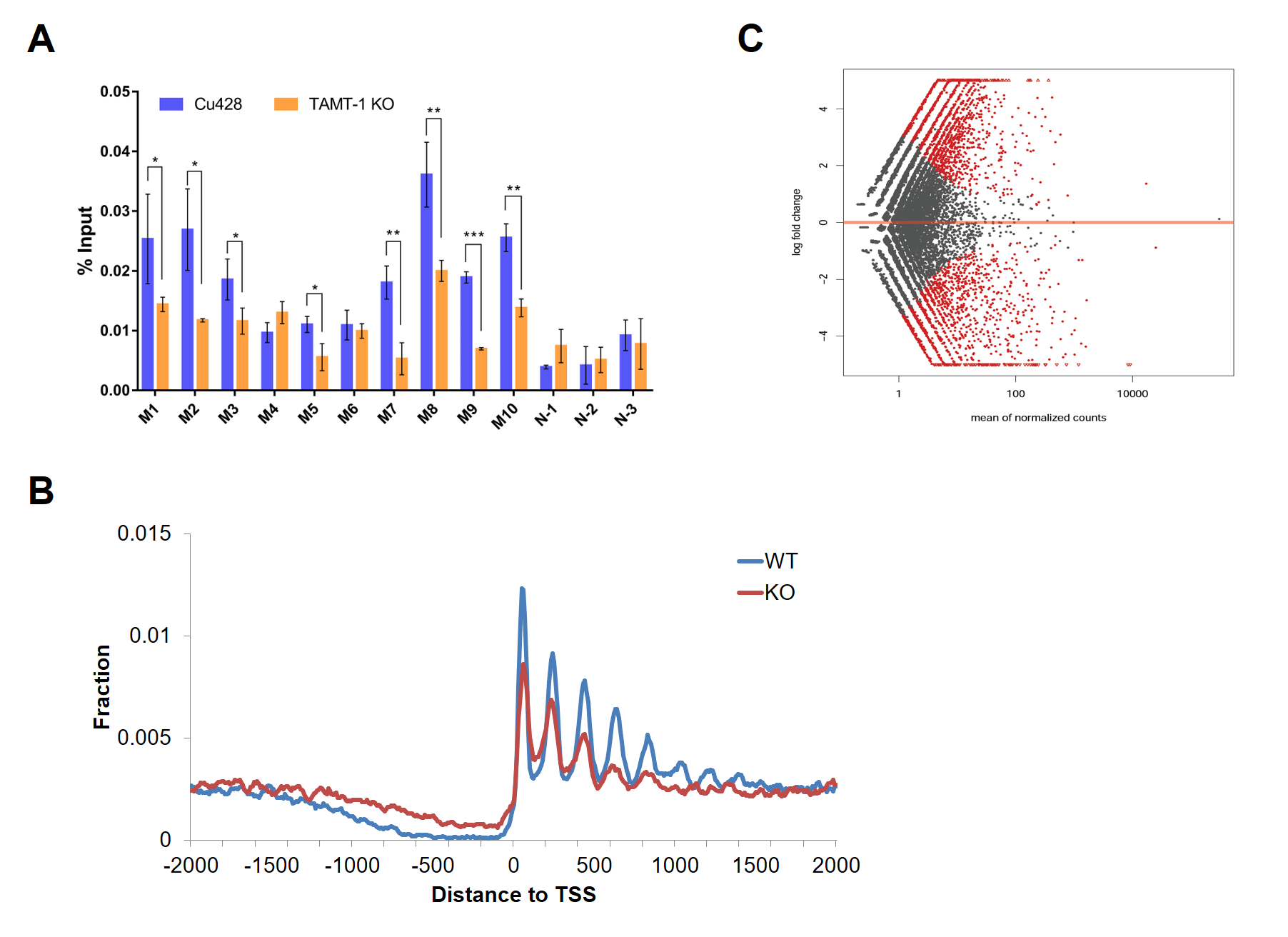


**Figure S8.** Effects of *tamt-1* knockout. (A) qPCR results of 13 selected 6mA sites validated by 6mA-IP-seq. gDNA was fragmented to 200 bp and was conducted to 6mA immunoprecipitation subsequently. qPCR was performed using specific primers covering these sites. The abundances of enrichment after immunoprecipitation were calculated by the percentage of immunoprecipitated products to input DNA samples (n = 6, mean ± s.d.). M1-M10: with 6mA peaks via 6mA-IP-seq; N1-N3: without 6mA peaks via 6mA-IP-seq. * p<0.05; ** p<0.01; *** p<0.005. (B) Nucleosome array of KO and WT samples. The positioning of KO is less sharp than WT in TSS adjacent region. (C) Differentially expressed gene analysis of *tamt-1* KO cells comparing to WT cells. Each dot represents one gene. Red dots represent significantly changed genes (p<0.05).
