# Supplementary material for: N6-methyldeoxyadenosine directs nucleosome positioning in Tetrahymena DNA
Source: Genome Biol. 2018 Nov 19;19:200. doi: 10.1186/s13059-018-1573-3 (PMC6245762; doi:10.1186/s13059-018-1573-3)
Supplement: Supplementary file 2 — Table S1. Genomic loci for individual 6mA testing. Table S2. Sequences of primers used for tamt-1 KO experiment. (DOC 47 kb) [file 13059_2018_1573_MOESM2_ESM.doc]

**Table S1. Genomic loci for individual 6mA testing**

| ID | Genomic loci | Gene | Genomic sequence (potential 6mA sites are marked in red) |
| --- | --- | --- | --- |
| M1 | scf_8253992:18,490-18,843 | TTHERM_01008630 | TTTACCTGTATTAACCGAAGGCTGCTAACTTAAACTTGGTCATTGTTTGAGTTGCTTCAATACCACCCTAAGCAACTATCCATTTTCTAGCCTTTTATAGAGCAGCTTCAATTTTAGGATTGTTGTTAATAGACTTTAAGTACCAGTAGTTAAAGACAGTAGCATCTAACTAACCAGTTTCAAGATTTTGTTCTCTGACTTGCTCCCATGAACCATCTTCGAATTAAGTCTAGAGGAGACGTTCTGTAAAGTATGTAGCGTTGAAAGCAGATTTATGAGTCCAATTCAAGACGAGTAACTCGAAGTAGTATTCACTGATAAACATTTCGCCTAAGTAAGGAGGATAGTACCAA |
| M2 | scf_8254464:363,129-363,463 | TTHERM_00580360 | ACTCTTAACTATAAGTGTCTTTAATTATAGGCCCTACAAGTATACCGTTTGGAGCTAATTAATGTATAAATTTGTTAACAGCCTTGAGAGTTGCTGCAGCTCCAAGGTGGATAATATCAAATGGAGCGTAGTCTTCTAATCCTTCTCTTCCATCTCCTTTTACCAAGACAATTTTTCCTGATTCAAGCAGTTCTTTATGATTTTTCATTATGTTCTTCTTAGAAATGTTAAGAACTCCTTATAAATGATCTAACCCATAGCATATTGCGCTTTCATCCTTCATAAGCTTTGCAAGTGCAGTTGTCATCCATCCGCTTCCAATACCAATATCTAA |
| M3 | scf_8254444:291,934-292,320 | TTHERM_00585250 | TATTCGGAACAGTTATATAGCACGGAAAGATGGATAAGACTGTCACTGTAATAGAATAATTATCAAATAAGAGATAGAAATATTAAATTTTTCTTCTTCTTATCATAATAATTTGAAAAATTTCATTAAAATCATTTAGAAATAGCACAAATTCTAAAAAAAATTTTCTATAATTATTTTAGCTCTAAAATTGCAATTGAAATATATTAATGAATTGTATTGCAGCATACTACTTTTTATATGGTTTAACAATTTAAATTTTAATTTGCATTTTTAATTATTAGGTTAAGGTTGATTACAGATATTGGTATCCTAAAACAAAAGAATTTAAATCAAAAACCAGCAAATATCAAGTTCATGATTCTGAAAACTTTTGCGTTACTGGA |
| M4 | scf_8254589:107,550-107,900 | TTHERM_00997670 | ATAAGGAGTCAGGTATGCTCCGTTTAGATAGAGTTCTCTTTTCAGCTGTTCATTACCCCGCTAATTATGGTTTTATTCCCTAAACCTACTGCGATGATAAAGATCCTTTGGATATTTTAGTACTCTGCTCAATTAACGTCGAGCCTCTTTGCTTAATAGAATCTAAAGTTATTGGTGTTATGCATATGATTGATGGAGGAGAGACTGATGATAAAATTATTTCTGTAGCTGCTCATGATGCTTCAGTAAATCACTTTAATGACATCAGTGAGCTACCTCAACATATGTTAATGCAAATCAGAAGATTCTTTGAAGACTATAAGAAATTAGAAAAGAAACAAGTCATTGTT |
| M5 | scf_8253899:58,881-59,231 | TTHERM_01222600 | AAGCAGCTTCTTTTGGAGCTAGAGTTGGATTAGCAGATTTTGTAAAGCCTAGTCCATAAGGATCAACTTGGGGTTTAGGAGGTAAATAAACAAATAATAAGATTCAAAAATGGCAAAATTAAGTAATTTGTAAATTAATAACAAATAAAAGGAACTTGTGTAAATGTTGGATGTGTGCCAAAGAAAATGTTCCACTATGCTTCTGAATTCGGAGATATTTTAGAGCACTAAAGAAATGCTGGTTGGGAAGTTCCACATAACATCAATCATAACTGGAGTACTTTAGTAAATAAGGTTTAGACATACATCAAAAGGTTGAACGGCATTTATATGGATGCTTTGAAAGACAA |
| M6 | scf_8254811:170,083-170,443 | TTHERM_00070750 | AGTAAAAGTGAGTCGAAGAGATATGAATACAACAAATGGTGGTGCTAGCACCTCATGCTCCGAAGATACTCCACATGAAAGAGTTTATACACCGCAAACATCGCACATAGTTACATAAGTCGAGCCATCGTTTTTCGATGATCTAGCTAAGGACTCGTAACTCTGGATTTCAAATCCTTGGGAGCATCCAAAAATCCGTAAGATGAATTTATCCAAGGTTATTCTACACGAAGGTCACCTTTTGAAGAAATCTAGAGACGAATAGCATATGTTTAAAGCCAGATATTTTGTGCTTTATGAAGATAGATTAGAAGTTTTTAGAGTAATTTAAAATTATTTTTAGTTGATTTAGCATTTTTA |
| M7 | scf_8254811:873,900-874,262 | TTHERM_00059140 | ATTTGTAATCATAACCTTATATATTTGTAGAATACTACCCACTTAAAGAAGAAGGATTCACATTACTATTTGCATTATAATTTTCCGTAACTCCTCCGTATAATCCAGAAGGGCTAGTATACGATGGAACAGGAGTGTTAGCTGTTTAGATTTACGAAGTGTAAGTTGCTGTGTTGATTTGTCCAGCGTTATTGCCTGCTGGAGTAATACCTAATAATTTTTAGATTGCTATGGAAGATTATAATTGTGGAGTTTAATCCTTTAAGCTGAATTTATCTGCTAATGAGTTTCTCATTTCAGATTTTTCAGGTCCTTGATGCGAAGAAGATATTCTTACTTTCTAAGACTGTTAGACAGGAGAT |
| M8 | scf_8253887:346,582-346,962 | TTHERM_00647500 | AGAGCTTTTCTATAACCTTAAAATGTTATTGAGGAAAGATATTTTAAACATTCATTAATAATAGCTGCATTTTCTCTTTCTAAAAGACCATCTTTCTTACATATTTCGTGTGTGAGAGTAATAGAAATGTTGTAATTACGTTTCTTACATTCTTCCTAAACTATCTCTCTGGCCTTGATTTCTTGAGAATTATTCATAGTAGAATAAACACCACTGATAACGAGGTTATGGATGTCTTATGCTATAATATCTTCTAAACTTTCTATTATTTCTTTTTCATTCATTTTTGTGTACTCTTTTTCTCCATCAAAGTAAAAACCACCATTCAAGATATAAGATGGTCCTTAAATTCTTTAAAGCAAATCAGAGTTTTCAATTTA |
| M9 | scf_8254638:604,449-604,836 | TTHERM_00190940 | CTGAAGCTATTTAGCATATAATGAATTTGTATGATTAAAGAGGAGATATTCTCTTTAACAAAGTTTCTACAAAATTCACTAGAATAAAAGATTTGATAAATTACACCAAAGGATAGATAGATATTCTACTTGATGGAATTTGTGACATCGAAAAAGAAGCCCTTGGCAAAGTATAGTATATAATTATGGACAATCATCCAAGGCATGGATTACCAGAAGAGGCATTGCAGAAACTGCAAAATTTTTAATAAAATAAACTTAATTTAGGATTAGGAAAATAAAAAAATTCAATAAGCAGCAAAGCCTTAAATAATTAATGTGAAGAAATCATGTAAGAGTTAGCTGATGTTACGTTAGATGTAATTGATAAAAAGATTAACACTTTTG |
| M10 | scf_8254582:312,385-312,718 | TTHERM_00138080 | CCGGTGGTCCTGGTGGCAAATTCGGTGGTGGCAGACCCGGTGGCCCTGGTGGCAAATTTGGTGCCAAGGGTCCTAGAGGTCCCAAGACTATTATTGTTAAGCATAGATTAGAAGGTGTTTTCATCTGCAAAGGTCAATAAGAAGCTTTGGTAACTAAAAATTTCTTCCCTGGTGAATCTGTTTACAATGAAAAACGTATGTCCGTTGAAGAAAACGGTGAAAAGATTGAATATCGTGTCTGGAATCCCTACAGATCTAAAATTGCTGCTGCTGTTGTTGGTGGTATTTCTGATATTCATATTAAACCTGGCTCCAAGGTTCTCTATTTGGGTA |
| N1 | scf_8253992:16,898-17,301 | TTHERM_01008630 | TAATAGTCCCCATGAGGTTTGAGTGACAGTAGATTTACCTATACCATTCCACTTTTCAGGATCATTATAAGAAAGGACACATTCACCAAAGCCACCATCTTTATTTTATTTATTAAGCAAATAATTTATACTATTTTGTACCCACTGTTCATTTAAGTCGTAATTGACTCTTGCTAAACCTGGTACAACAGCTCCAACAGCCATAATGTAATTTACACCCCATCTAGCTTACCAAGATCCCCACTTGTTCTAAGTCTTTCTTTGATATTTAATCATATTTTAAATCTAAGGATGATTAGCTTGATATCCAAACTCACCCAATCCTTCCATGATGTGACCAGTAATATCAGGACATGAGGGATCGAAGATTTCAGCTGAGTTAGCAATACCAGCCATATTGAAG |
| N2 | scf_8254464:361,963-362,298 | TTHERM_00580360 | TTTCTCTTCAATTTAAATATTTTCTTTTTATGTTTGTAATTTAATATGAAATTAATAACAAAACCTGTAAATTAATTTTTACAATTTTATTTGTTTAAATTTAACACAAAAAAAAAATATTTTTTTAATATTTAAATGAATTTCATATTTATTTATAATAATTATATAAAATTAAAATAATTATGAATTAAGTTATAAAGTAGACAACAATTTATATAAAAACATATTAAATTTTACTTTTAAAATTTATTATTATATAAAATCACTTTATTTTGAACAAATCACAGAAATAATTGTTAAGAGAGCACAATTAAAATTTCAAACTTAGTTTTCAT |
| N3 | scf_8254444:293,002-293,386 | TTHERM_00585250 | AGACATAATTGTTTTTAGTTTTCTCAGTTAATATAAAATAATTTCAATTCTTAGTTTACTTTTCATGTTTTATATCACTTTTTAAATTATTTTTATTAGCTTATCAAAATAAAAATGTTATATTTTAATATTTATTTCATGAAATGCTATCAAAATAAATGTATTTATCAAAAATATTTTTTGAAAATATCTACTATTTATTTACTTTTTCTTAAAAAAAATATATTTTTGACTTTTAATCAATACTGACTAACGTTAAATAGCTTTAAAAAAATATATTTTCTAACATATATTATAAATTTAAATGGTAAAATAAATTTCTATGTTATATATAATTTTTTGAATTTAATAATTCATATATATTTATCACTTTATAATGGTAGC |

**Table S2. Sequences of primers used for *tamt-1* KO experiment**

| **Description** | **5’-sequnce-3’** |
| --- | --- |
| tamt-1 KO 5'UTR Forward | GGGAACAAAAGCTGGAGCTCCATCTAATTAAGTTAGTTATTATTTAATTAATTTGT |
| tamt-1 KO 5'UTR Reverse | TAAATTGTATATCGAATTCCTGCAGTTACCTTATTATTGCTAATACTCCCTT |
| tamt-1 KO ORF Forward | TTTACTGGAAAAATGCAGCCCGGGGGATCCAACCTATTTCTACTCTAAACCCT |
| tamt-1 KO ORF Reverse | TCGAGGTCGACGGTATCGATAAGCTTGTTTTATGATTCTACCCAGCAGT |
| tamt -1 RT-qPCR Forward | GGTGAAACTTACGAAAAATGGATGC |
| tamt -1 RT-qPCR Reverse | GGTTCAGCTTCACAACGATTACAAC |
| ATU1 RT-qPCR Forward | CCTCCCCCTAAGTCTCAACC |
| ATU1 RT-qPCR Reverse | CGAAGGCAGAGTTGGTGATT |
